# LCOR Reverses Immune-Checkpoint Inhibitors Therapy Resistance Out of IFN Constraint in Triple-Negative Breast Cancer

**DOI:** 10.3389/fonc.2022.911572

**Published:** 2022-07-13

**Authors:** Jialin Zhou, Chun Feng, Kai Huang

**Affiliations:** ^1^ School of Medicine, Shandong University, Jinan, China; ^2^ The Center of Rehabilitation Therapy, The First Rehabilitation Hospital of Shanghai, Rehabilitation Hospital Affiliated to Tongji University, Shanghai, China; ^3^ Department of Medical Oncology, Qilu Hospital, Cheeloo College of Medicine, Shandong University, Jinan, China

**Keywords:** Immunotherapy, LCOR, triple-negative breast cancer, interferon, mRNA vaccine

Immune checkpoint inhibitors (ICIs), including anti-programmed cell death 1 (PD-1), anti-programmed cell death ligand 1 (PD-L1), and anti-cytotoxic T lymphocyte-associated protein 4 (CTLA-4), are revolutionary progress in cancer therapy. After the anti-CTLA-4 antibody ipilimumab was approved by the US Food and Drug Administration (FDA) for the application in treating advanced-stage melanoma, other mono antibodies targeting immune checkpoint proteins, such as nivolumab, pembrolizumab, durvalumab, and atezolizumab, are authorized and used in the treatment of multiple cancers (1, 2). ICIs significantly extended overall survival (OS) and have been the first-line therapy for certain patients with advanced non‐small-cell lung cancer (3). In metastatic melanoma, ICIs and targeted therapies provided increased five-year progression-free survival rates and five-year OS (4). Patients who have breast cancer, especially triple-negative breast cancer (TNBC) lacking estrogen receptor (ER), progesterone receptor (PR), and human epidermal growth factor receptor 2 (HER2) benefit from pembrolizumab monotherapy and atezolizumab monotherapy significantly (5, 6). TNBC is the most aggressive breast cancer and has a high incidence in young women. KEYNOTE-012, a phase Ib trial, evaluated the safety and efficacy of pembrolizumab in TNBC patients, showing an overall response rate of 18.5% with 56.3% of the patients suffering from immune-related adverse events (irAE) (7). KEYNOTE-173 trial assessed the effectiveness of pembrolizumab plus chemotherapy in patients with TNBC in the early stage, and reported promising efficiency against tumor progression, with manageable toxicity (8). A meta-analysis of neoadjuvant chemotherapy in combination with ICIs also suggested the treatment option elevated clinical outcomes of TNBC patients (9). Although ICIs plus chemotherapy repeatedly showed exciting efficacy, only a relatively small proportion of the patients had a positive response to it. More innovative combinations of ICIs with chemotherapy or radiotherapy are in development (10). Meanwhile, researchers have been attempting to explore biomarkers related to the outcomes of immunotherapy in breast cancer. The currently confirmed biomarkers include PD-L1 expression and tumor-infiltrating lymphocytes (TILs) (11). A study researched the relationship between TILs with response to neoadjuvant chemotherapy in breast cancer and reported that the pathologic complete response (pCR) rate was positively relevant to TILs in human HER2-positive and TNBC samples (12). However, many challenges still exist in cancer immunotherapy, among which ICIs resistance is an important issue to be settled (13). ICIs resistance includes intrinsic resistance and acquired resistance. The former one appertains patients who have no responsiveness to ICIs initially, while the latter is defined as when patients primarily benefit from ICIs at an early stage while diseases progress during ICIs therapy (14, 15). Several essential mechanisms account for intrinsic or acquired resistance, including deficiency of tumor antigenicity, interferon (IFN)-γ signal pathway, and antigen presentation machinery (APM) (14, 16). IFN-γ is secreted by the T cell and binds to its receptor, thus activating Janus kinase (JAK), which induces PD-L1 expression in the tumor cell. In some scenarios, PD-L1 expression is decreased due to mutations in JAK. Therefore, the effect of IFN-γ is less significant (16, 17). APM includes multiple proteins, including β2m, HLA genes, and TAP genes. Ligand-dependent corepressor (LCOR) is a protein regulating cell differentiation in normal or cancer cells. In this study, Pérez-Núñez, Iván et al. confirmed that LCOR can activate APM through a mechanism irrelevant to the IFN pathway (18).

The researchers harvested tumor cells in mouse breast cancer models with ICIs resistance. Transcriptomic analysis of immunotherapy-resistant (IRT) tumors suggested that genes in APM and IFN-γ pathways are downregulated while CSC-like signatures represented by ES-1, NOS-targets, and breast-CSCs are enriched. Some stem cell transcription factors, especially LCOR, decrease in IRT cells. Flow cytometry analysis showed mammary stem cells (MaSCs) markers CD24+/CD29 hi increases in IRT cells compared to control. After coculture of CD8+ T cells and AT3-OVA cells under anti-PD-L1 treatment, survived cells are CD24 lo/CD44 hi AT3 cancer stem cells (CSCs). Next, the researchers showed that CD24 lo/CD44 hi cells are enriched in human CSCs from patients with TNBC. LCOR and APM-related genes like β2M, HLAs, and TAP, are down-regulated in CSCs compared to non-CSCs. APM gene expression is positively correlated with LCOR level. To investigate how LCOR regulates APM, the researchers build LCOR overexpression and knock-out models in MDA-MB-231 and HMLE cells. Although IFN-γ can improve LCOR effects on APM, the JAK1/JAK2 inhibitor ruxolitinib failed to alter the effect of LCOR on APM *in vitro*, indicating that LCOR can activate APM independently of IFN-γ. Chromatin immunoprecipitation sequencing (Chip-seq) analysis implicated LCOR interacted with APM by interferon-stimulated response element (ISRE) binding.

Then the authors tried to figure out the effects of LCOR-mediated APM activation. Coculture of AT3-OVA and Py8119-OVA cells with CD8+ OT-I T cells and 4TO7-EGFP with CD8+ JEDI T cells indicated that Lcor-overexpression (OE) cells receive more immune attack. AT3-OVA-Lcor-OE cells with siRNA β2M and AT3-OVA-Lcor-knockdown (KD) cells showed increased cell viability after 72h cocultured with OT-1 CD8+ cells. LCOR-KD cells decreased T cell activation while Lcor-OE cells induced it. Furthermore, CD45+ and CD8+ immune cells became multiplied in Lcor-OE tumors according to immunohistochemistry. Percentage of CD45+/CD3+, CD45+/CD3+/CD4+ and CD45+/CD3+/CD8+ lymphocytes is elevated in Lcor-OE 4TO7 tumors. Analysis of samples from TNBC patients confirmed that high expression of Lcor is related to CD4, CD8, and γδ T lymphocytes enrichment. The above results proved that LCOR can enhance T cell infiltration in TNBC against tumors.

The researchers then proceeded to evaluate the relationship between LCOR level and the responsiveness of ICIs in TNBC patients. In post-ICB samples, LCOR reduced significantly compared with pre-ICIs. In the TONIC trial and I-SPY2 trial, lower levels of LCOR are observed in non-responders to ICIs. In mouse models, over-expression of LCOR significantly reduced the growth rates of tumors combined with or without anti-PD-L1. In particular, LCOR-OE transplanted tumors are eliminated under anti-PD-L1 treatment. However, anti-CD8/CD4 treatment can promote tumor growth, validating that the responsiveness to anti-PD-L1 in the LCOR-OE tumors requires an adaptive immune system. These data demonstrated that higher LCOR levels and anti- PD-L1 exposure can enhance the adaptive immune system to kill tumors.

In the end, inspired by COVID-19 mRNA vaccines, the investigators proposed the strategy of mRNA-based LCOR therapy, by transfecting LCOR mRNA into tumor cells using extracellular vesicles (EVs). A preclinical lung metastasis model was designed to measure the effects of LCOR mRNA therapy. Mice administered EV-based LCOR mRNA therapy and anti-PD-L1 had lung metastasis regressed drastically and survival prolonged. These results indicated that LCOR mRNA therapy combined with anti-PD-L1 is one promising treatment option in the future.

This study demonstrated the crucial role of LCOR in regulating tumor immunogenicity and responsiveness to immunotherapy. In TNBC, the level of LCOR expression in CSCs is relevant to the therapeutic effect of ICIs. In another study, a group generated an mRNA vaccine covered by lipid-coated calcium phosphate nanoparticles. The complexes can be uptaken by dendritic cells and the mRNA inside can encode corresponding protein. The vaccine can enhance the antigen presentation system and activate T cells to attack tumor cells. Furthermore, a combination of PD-L1 siRNA and mRNA vaccine was shown to significantly decrease the expression of PD-L1 in the dendritic cells, which can promote an anti-tumor immune response (19). A previous study indicated that the variety of neoantigen-specific T cells was elevated with the application of a dendritic cell vaccine (20). Some researchers produced a vaccine against neoantigens. The vaccine can enhance T-cell responses significantly (21). Another preclinical study showed that the MUC1 mRNA vaccine plus anti-CTLA-4 antibody can improve the activity of immune cells in TNBC (22). These data support that LCOR is a potential target to enhance the immune response in coordination with ICIs. However, there are some concerns regarding the clinical application, including the safety of LCOR mRNA vaccines, the stability and immunogenicity of mRNA vaccine (23), and the side effects due to adaptive immune system activation. Nevertheless, this is an inspiring discovery and provided a potential strategy to improve the efficacy of immunotherapy.

## Author Contributions

JZ and CF wrote the manuscript. KH initiated the idea and edited the manuscript. All authors contributed to the article and approved the submitted version.

## Funding

This study was funded by the Taishan Scholars Program of Shandong Province (No. tsqn202103174).

## Conflict of Interest

The authors declare that the research was conducted in the absence of any commercial or financial relationships that could be construed as a potential conflict of interest.

## Publisher’s Note

All claims expressed in this article are solely those of the authors and do not necessarily represent those of their affiliated organizations, or those of the publisher, the editors and the reviewers. Any product that may be evaluated in this article, or claim that may be made by its manufacturer, is not guaranteed or endorsed by the publisher.
